# Supplementary figures and images for: USP30 sets a trigger threshold for PINK1–PARKIN amplification of mitochondrial ubiquitylation
Source: Life Sci Alliance. 2020 Jul 7;3(8):e202000768. doi: 10.26508/lsa.202000768 (PMC7362391; doi:10.26508/lsa.202000768)

Source Data Figure 2A

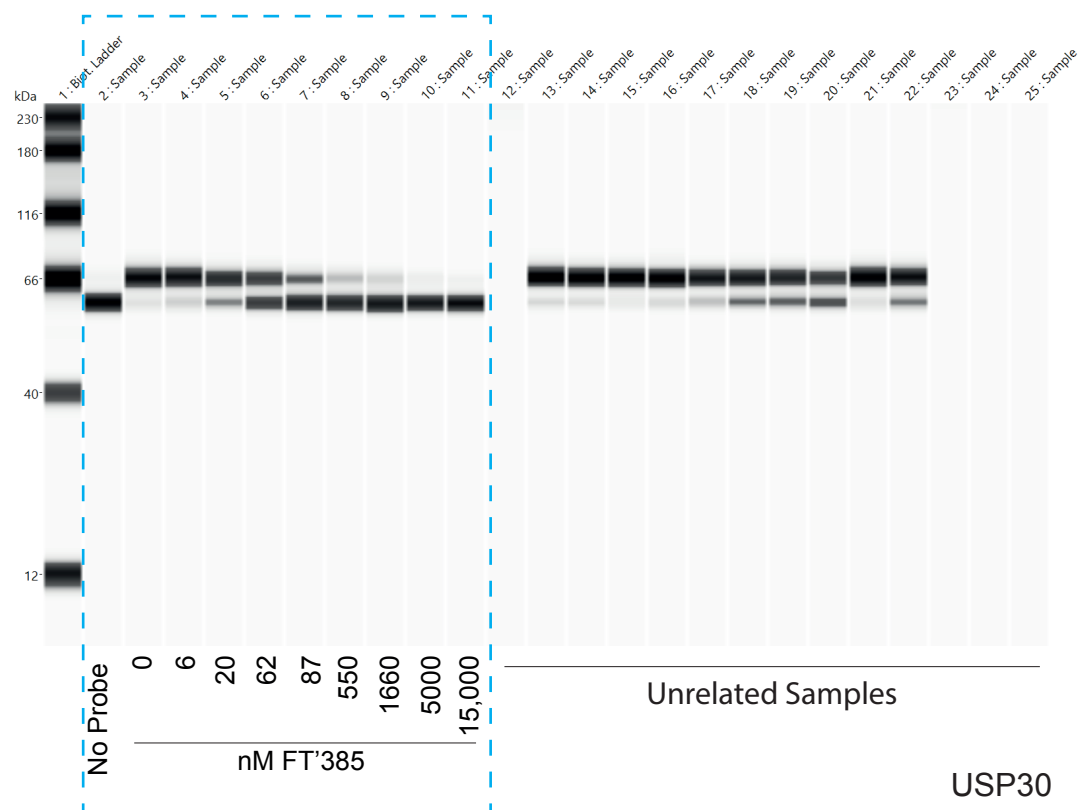

Source Data Figure 2B

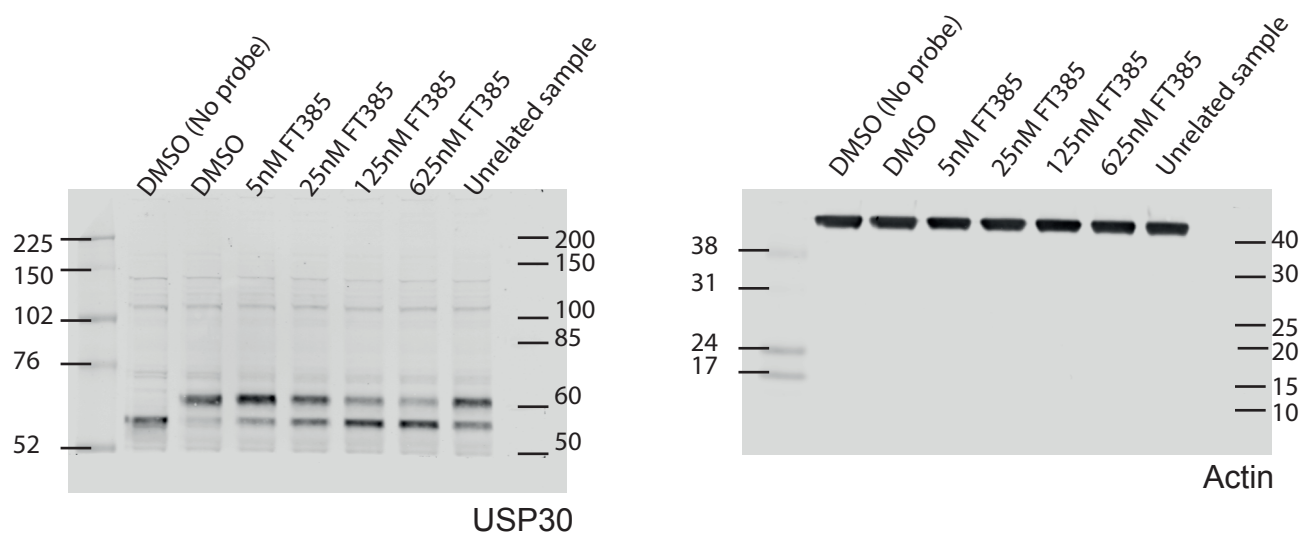

Supplement: Supplementary file 1 [file LSA-2020-00768_SdataF2.1.pdf]

Source Data Figure 5E

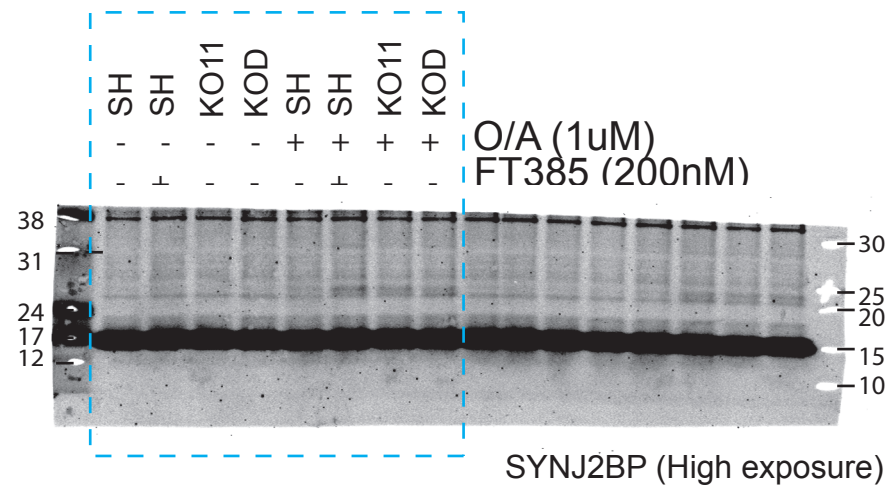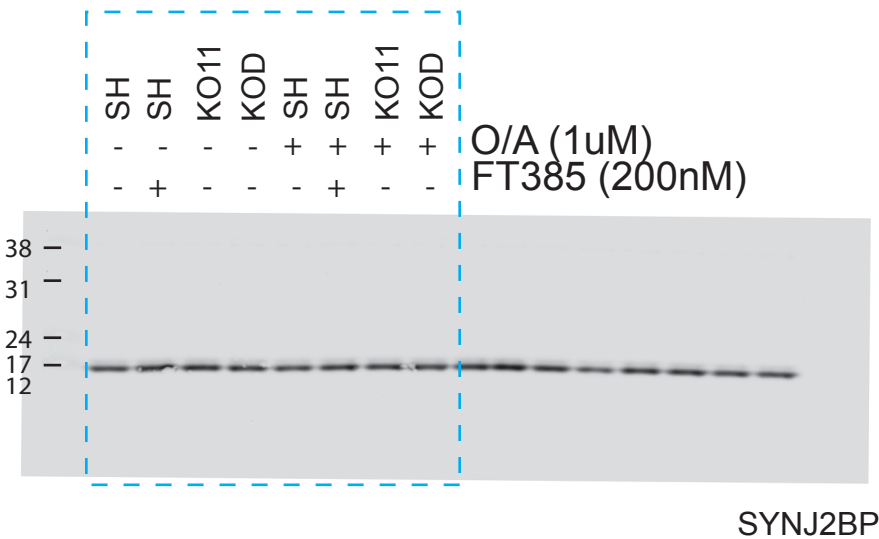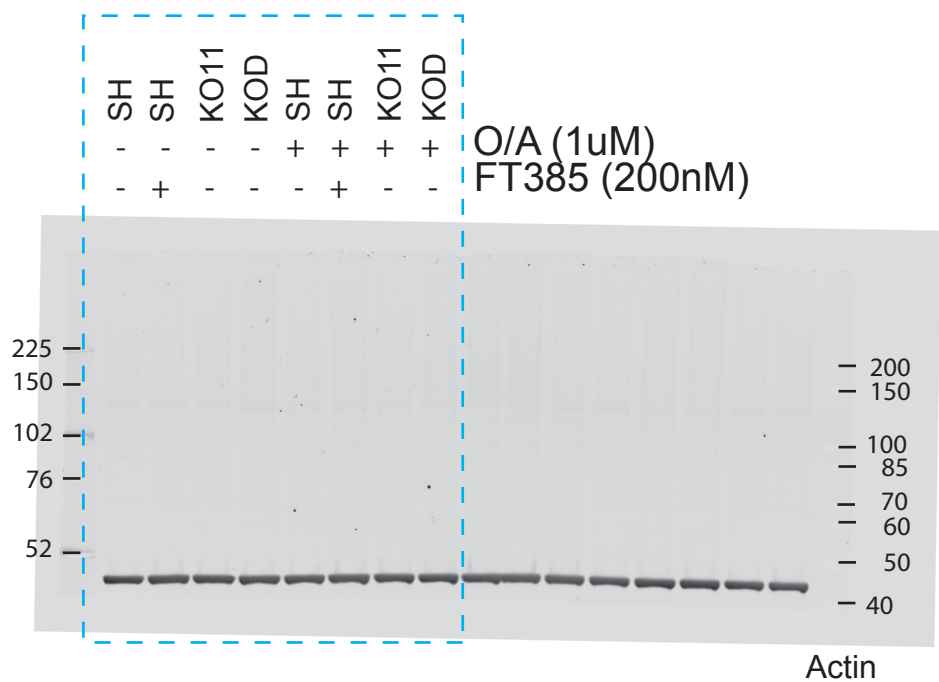

Supplement: Supplementary file 9 [file LSA-2020-00768_SdataF5.pdf]
