# Supplementary material for: USP30 sets a trigger threshold for PINK1–PARKIN amplification of mitochondrial ubiquitylation
Source: Life Sci Alliance. 2020 Jul 7;3(8):e202000768. doi: 10.26508/lsa.202000768 (PMC7362391; doi:10.26508/lsa.202000768)

Source Data Figure 3A

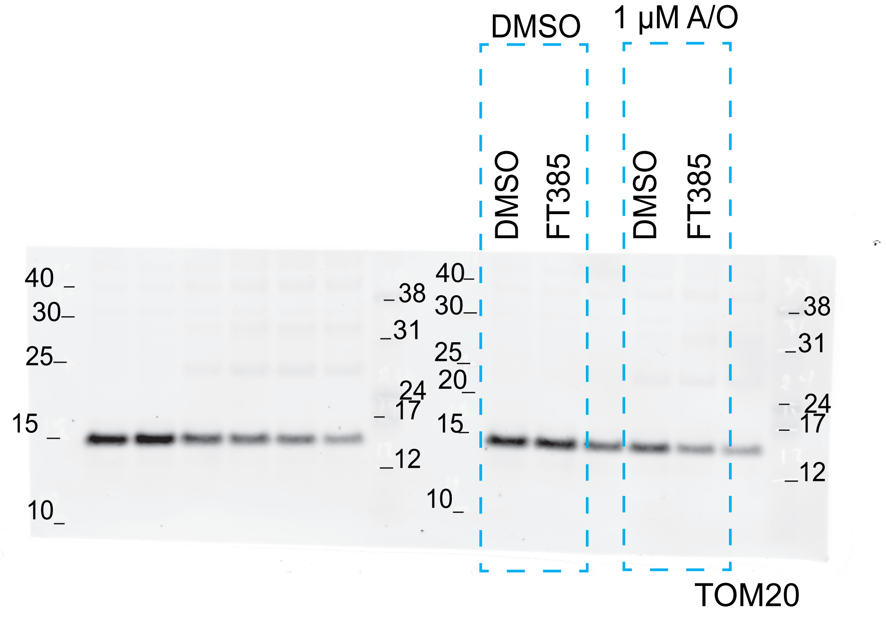

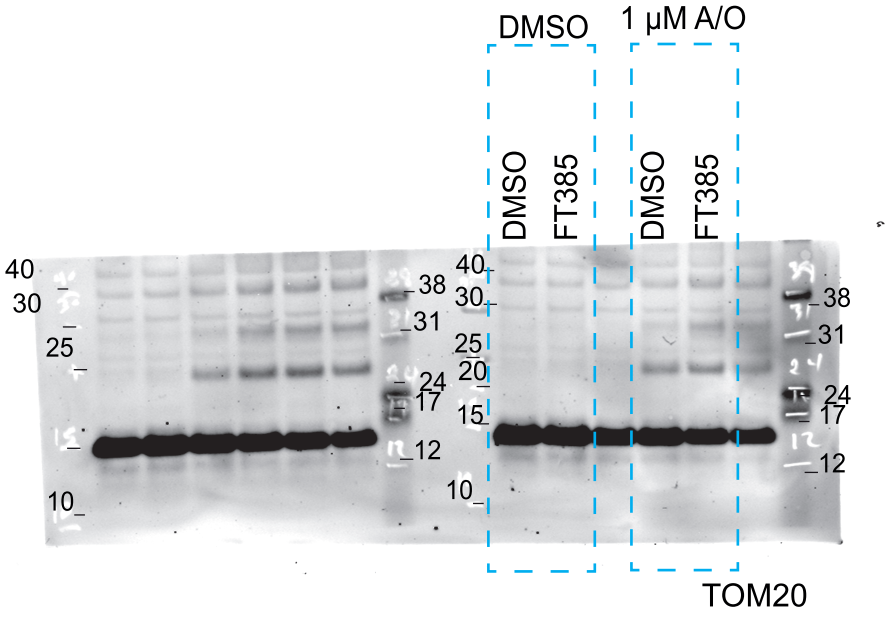

Source Data Figure 3A Cont.

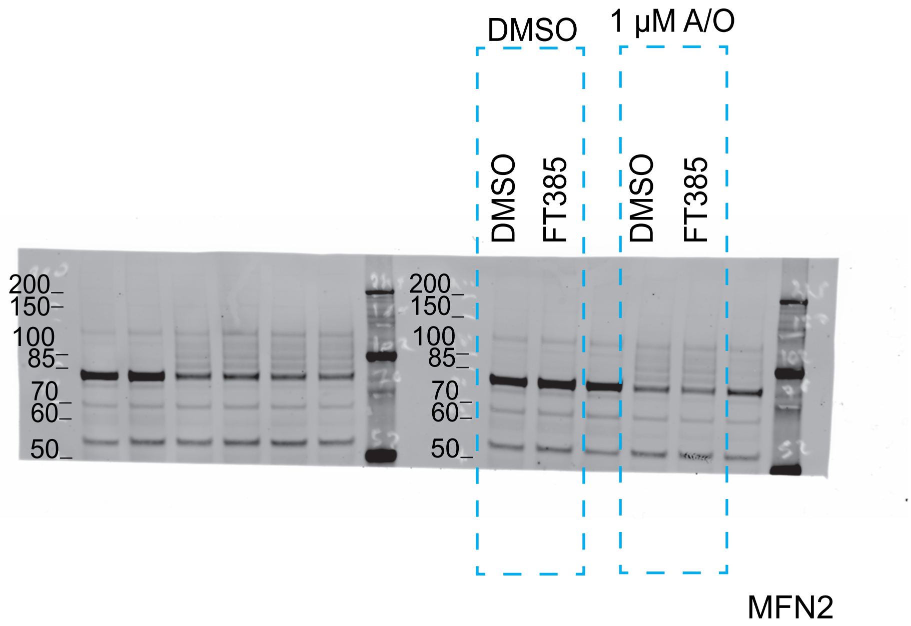

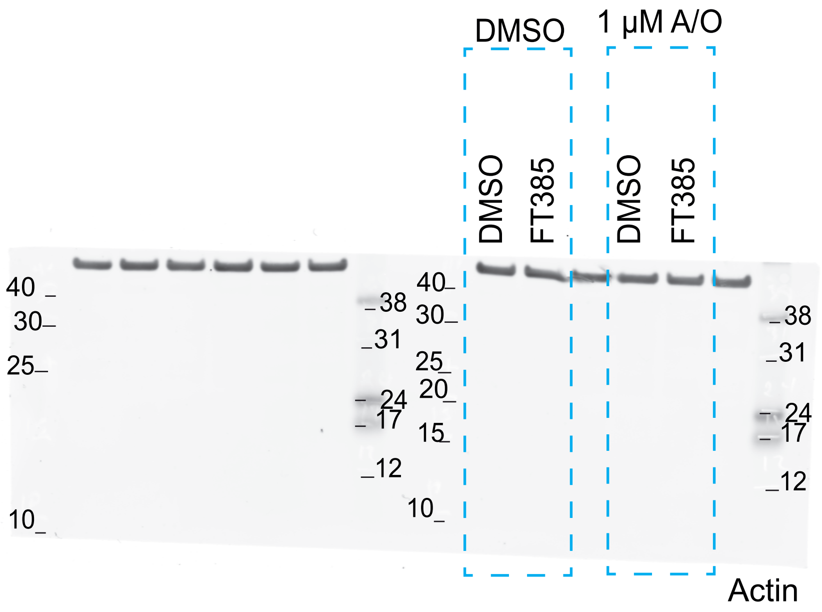

Source Data Figure 3A Cont.

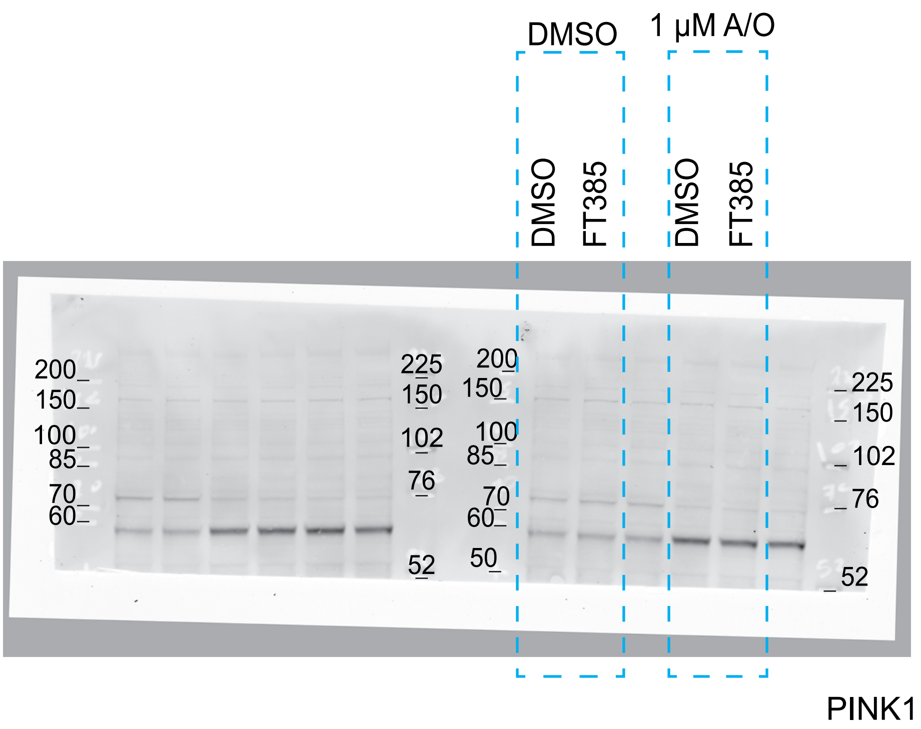

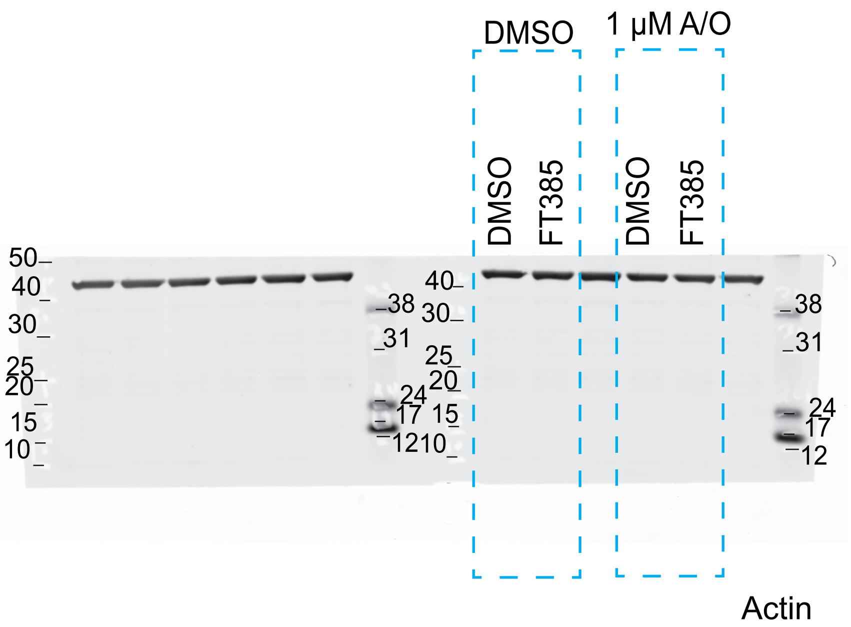

Source Data Figure 3B

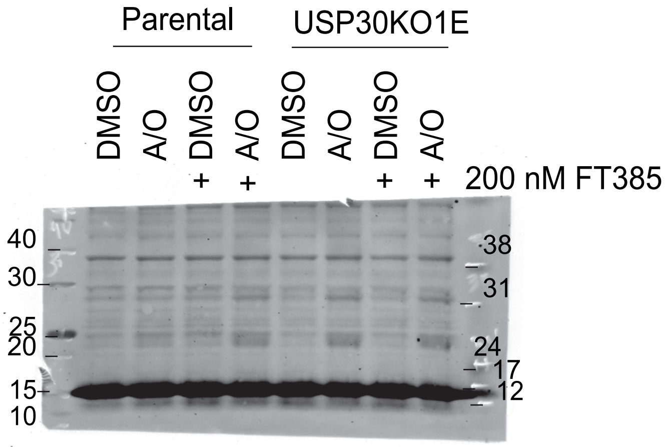

TOM20 (High Exposure)

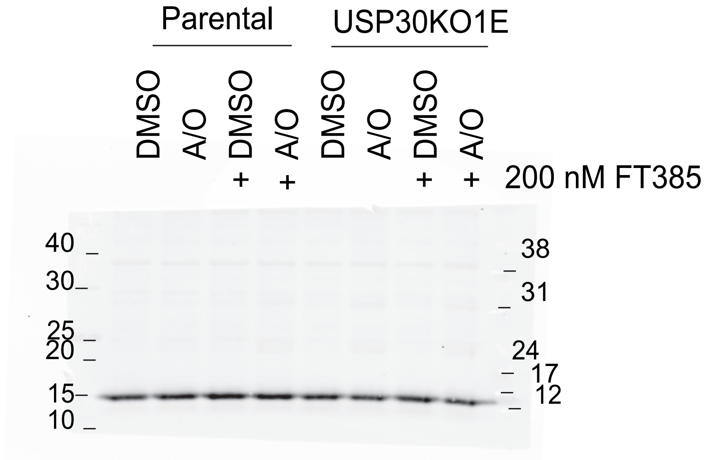

TOM20

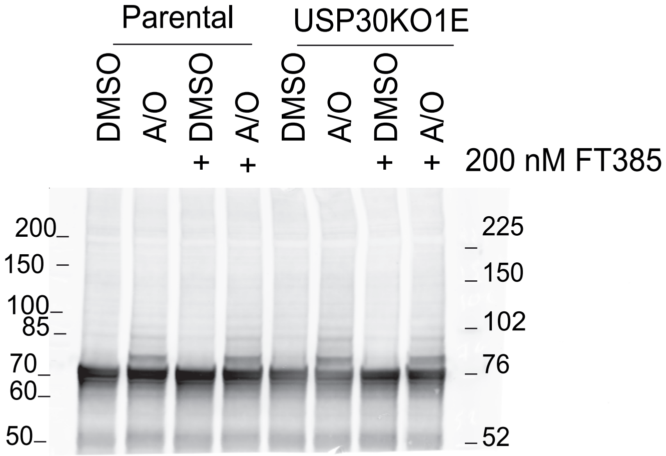

PARK2

Source Data Figure 3B Cont.

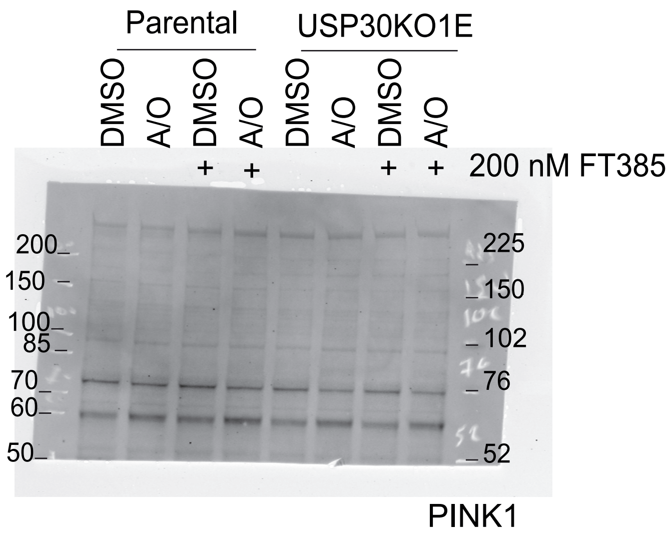

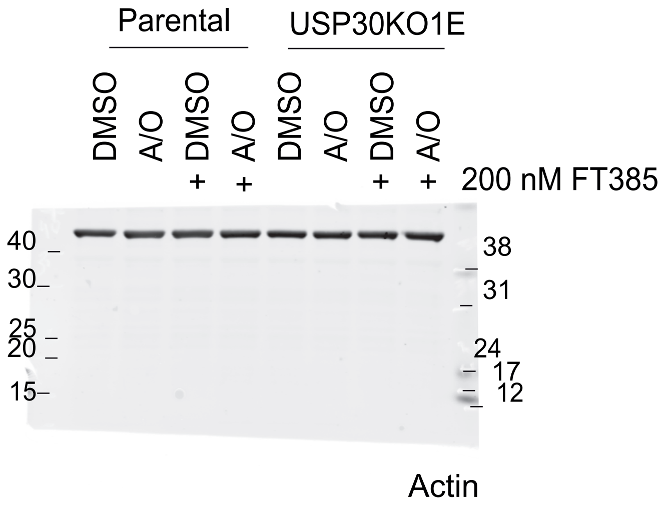

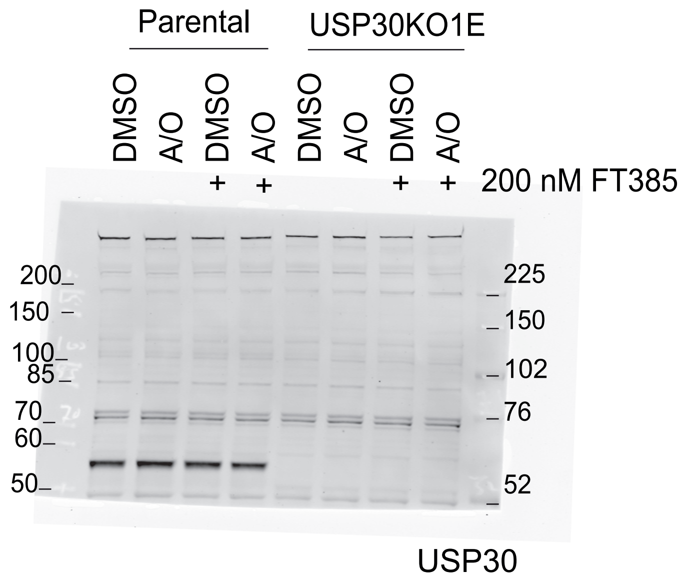

Source Data Figure 3C

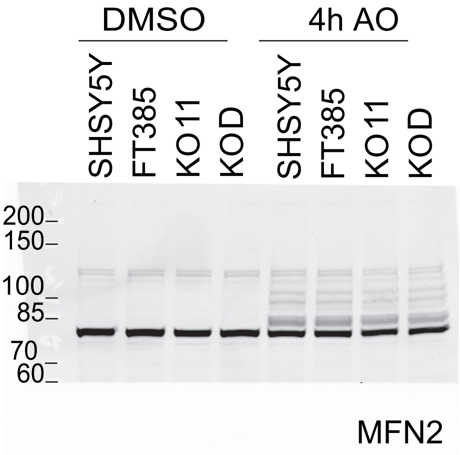

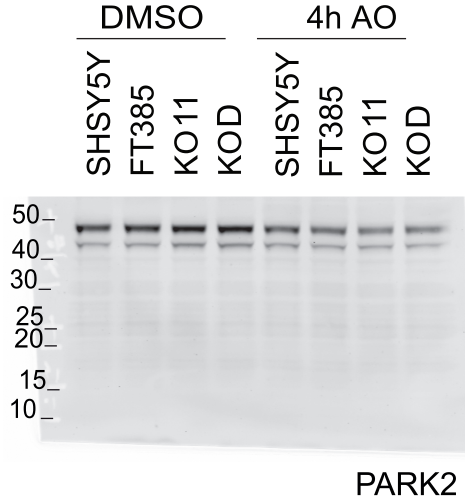

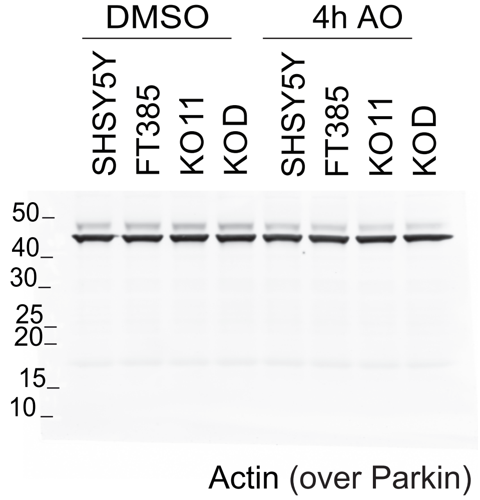

Source Data Figure 3C Cont.

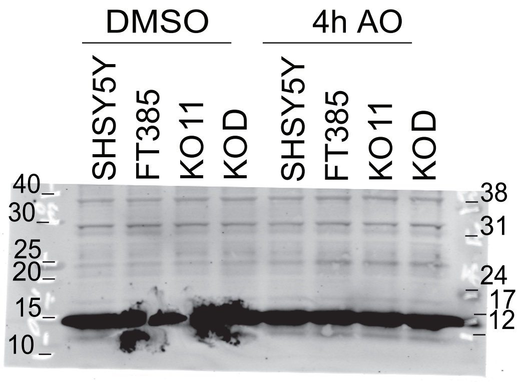

TOM20 (Higher exposure)

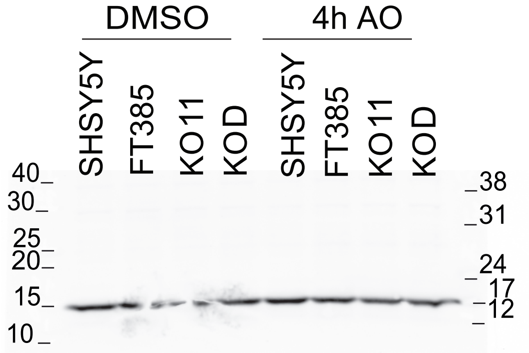

TOM20

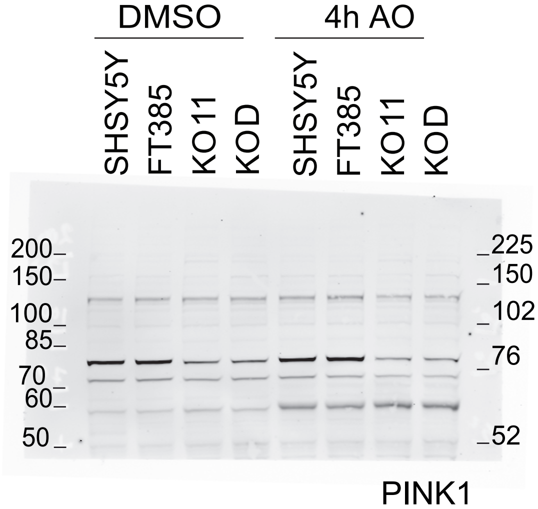

PINK1

Source Data Figure 3C Cont.

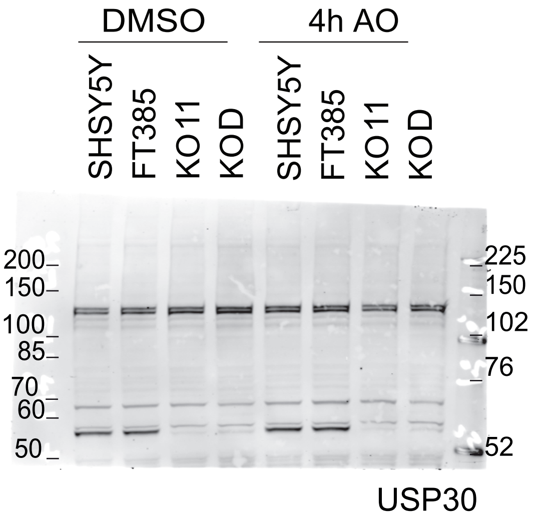

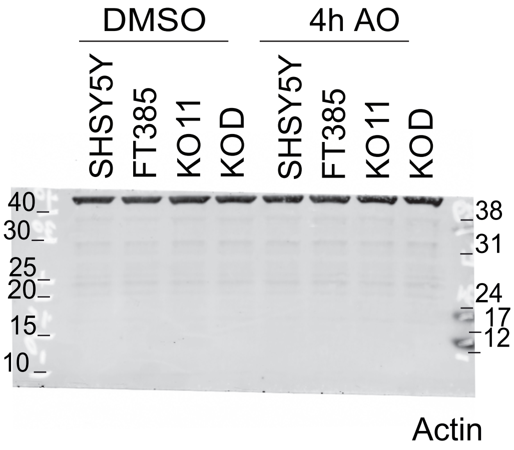

Source Data Figure 3D

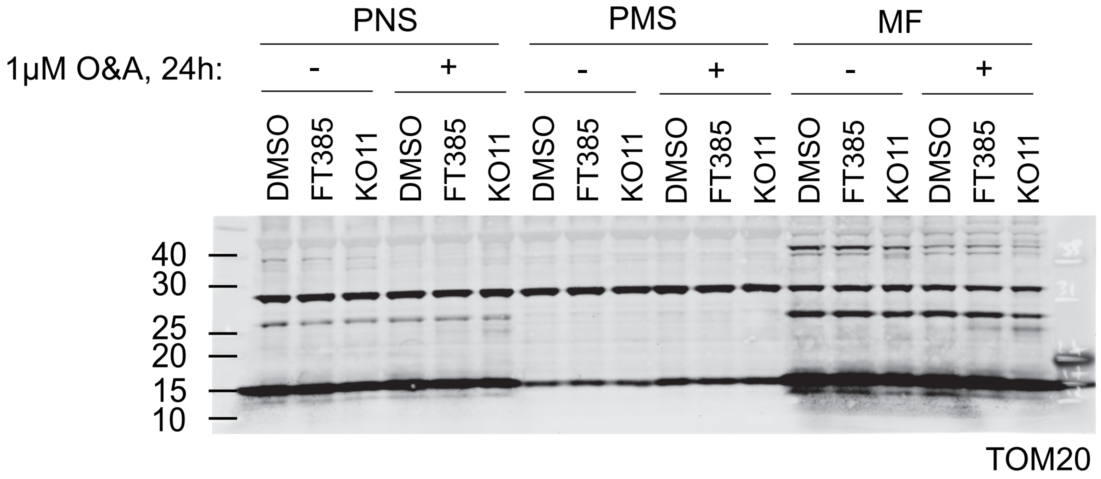

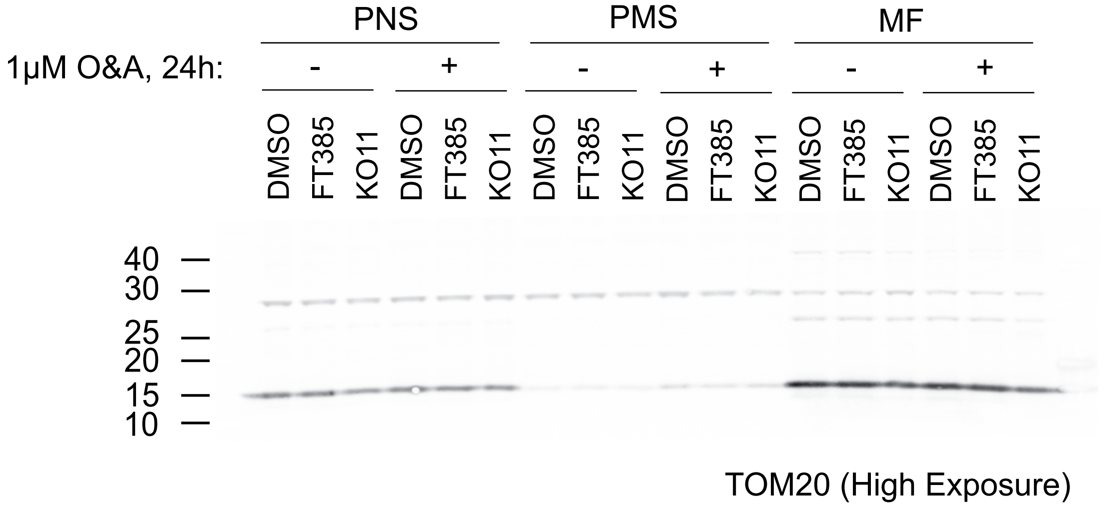

Source Data Figure 3D Cont.

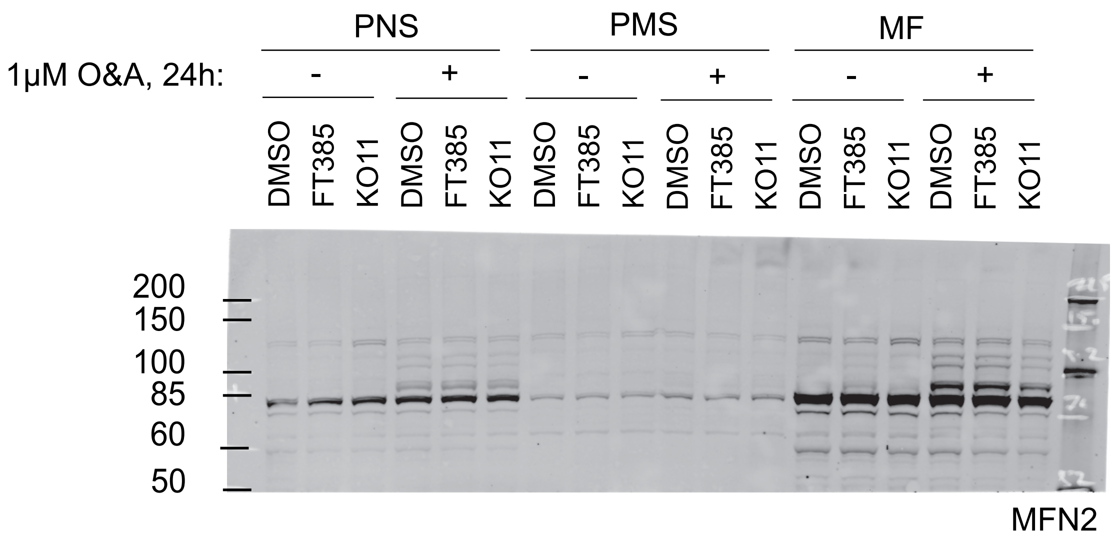

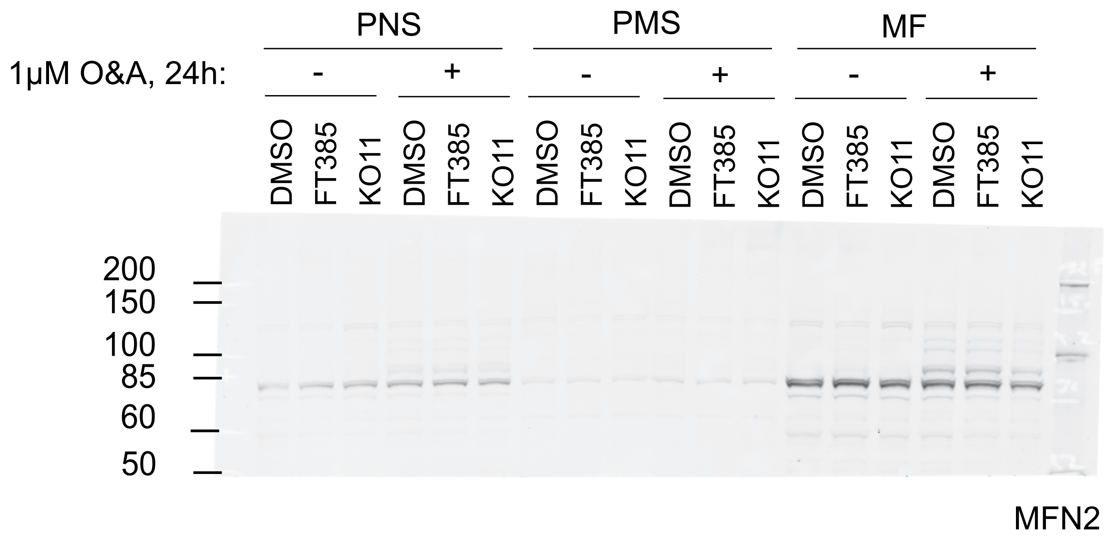

Supplement: Supplementary file 3 [file LSA-2020-00768_SdataF3.1.pdf]
