# Supplementary material for: USP30 sets a trigger threshold for PINK1–PARKIN amplification of mitochondrial ubiquitylation
Source: Life Sci Alliance. 2020 Jul 7;3(8):e202000768. doi: 10.26508/lsa.202000768 (PMC7362391; doi:10.26508/lsa.202000768)

Source Data Figure S2A

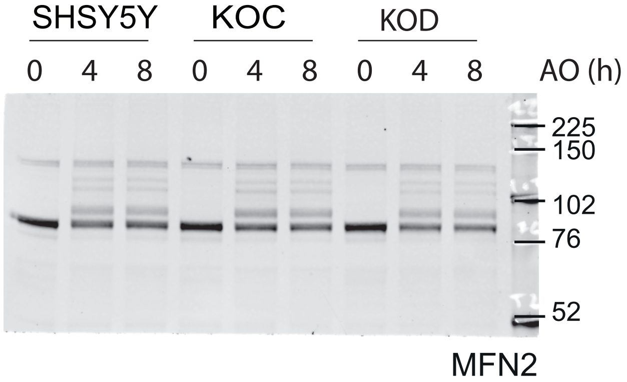

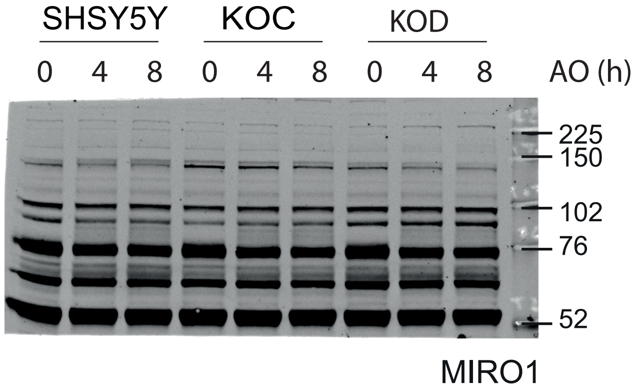

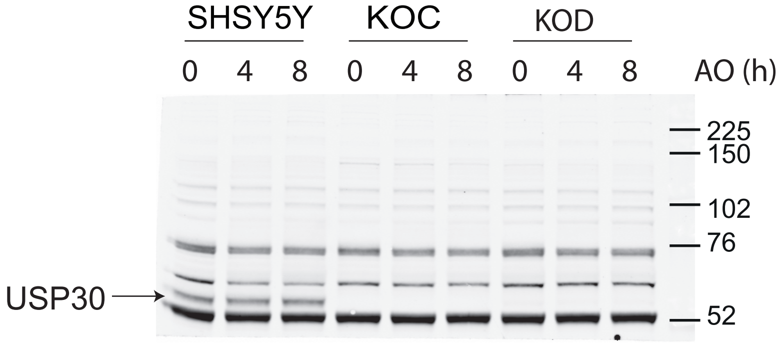

USP30 (reprobed over MIRO1)

Source Data Figure S2A Cont.

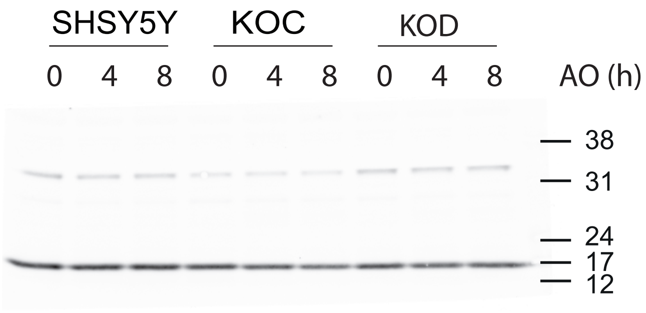

TOM20 (Lower exposure)

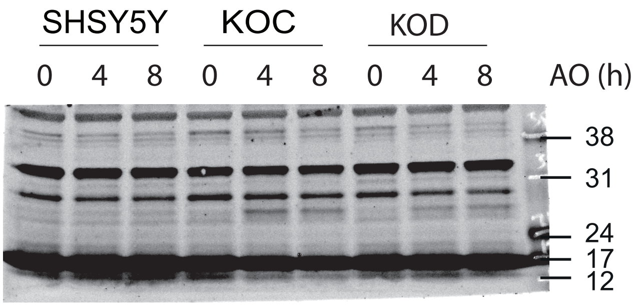

TOM20

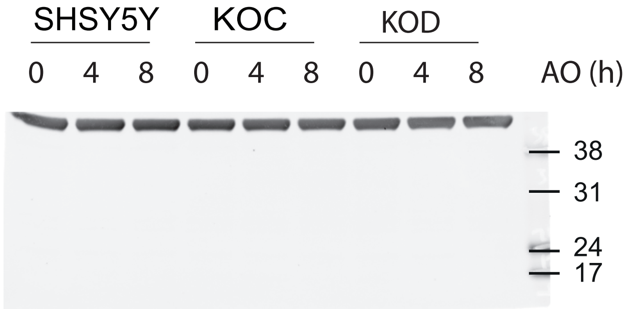

Actin

Source Data Figure S2A Cont.

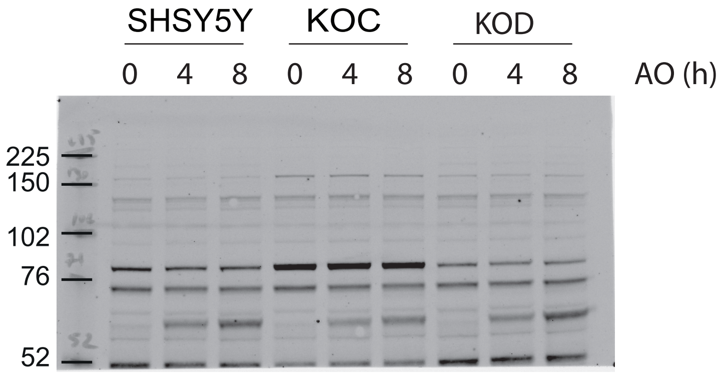

PINK1

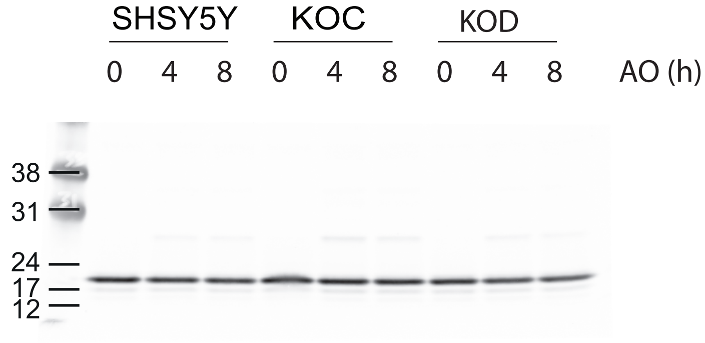

FIS1

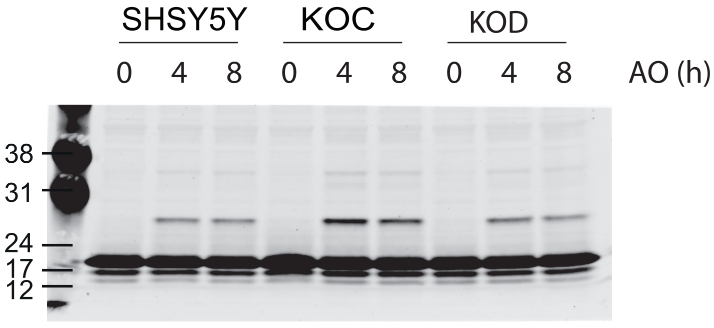

FIS1 (High exposure)

Source Data Figure S2A Cont.

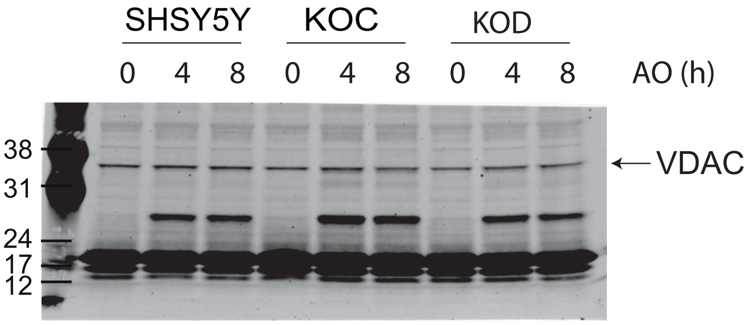

VDAC (reprobed over FIS1 Rb)

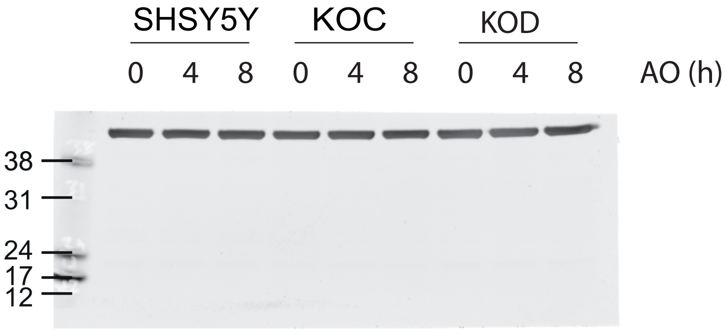

Actin

Source Data Figure S2D

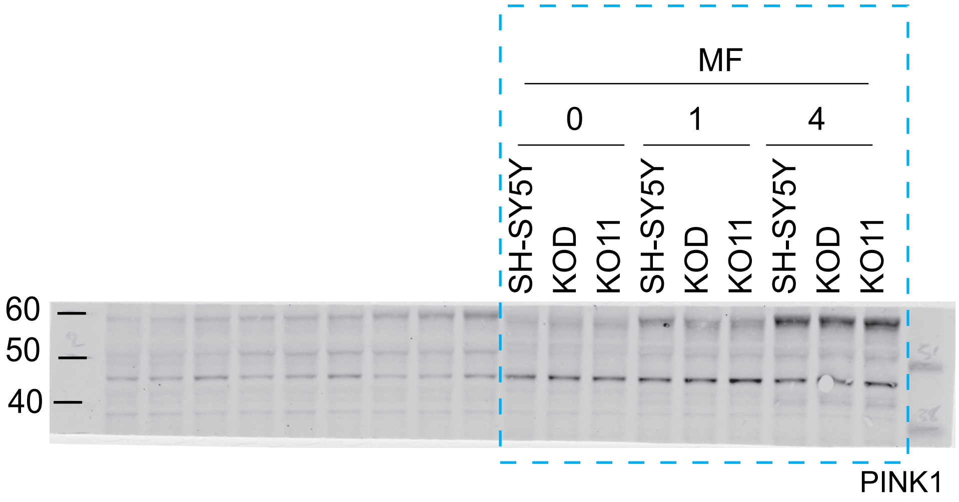

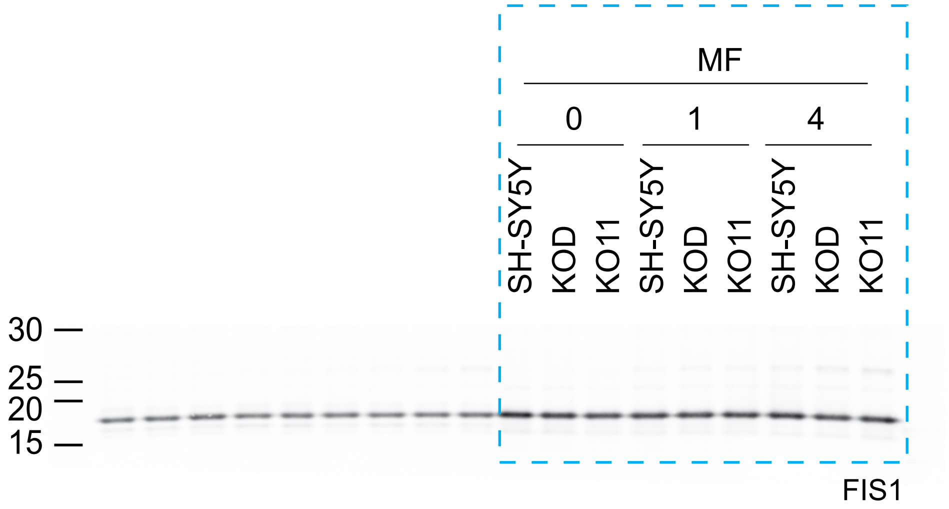

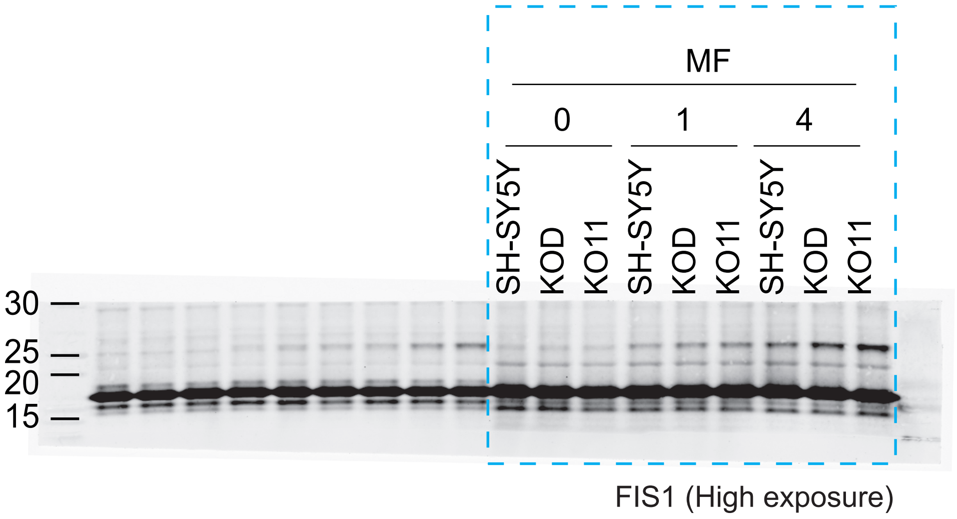

Source Data Figure S2D Cont

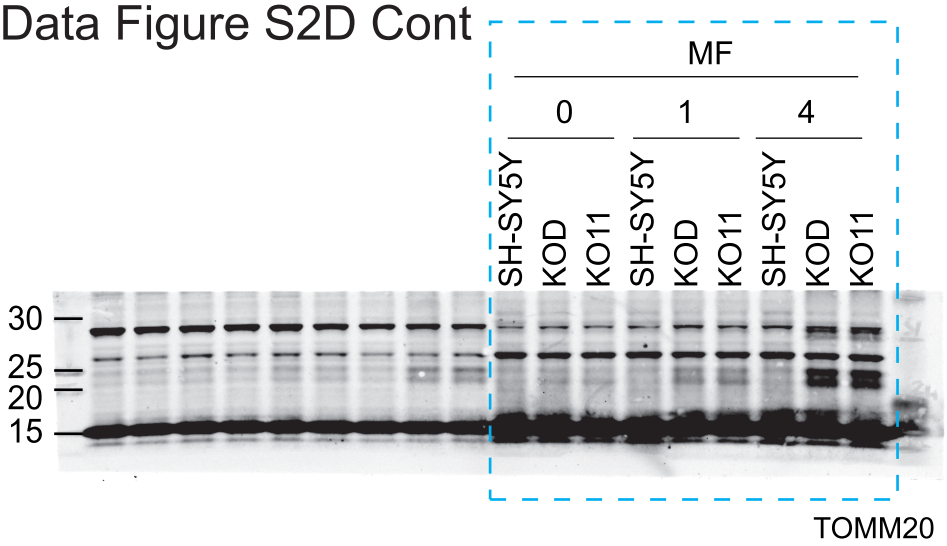

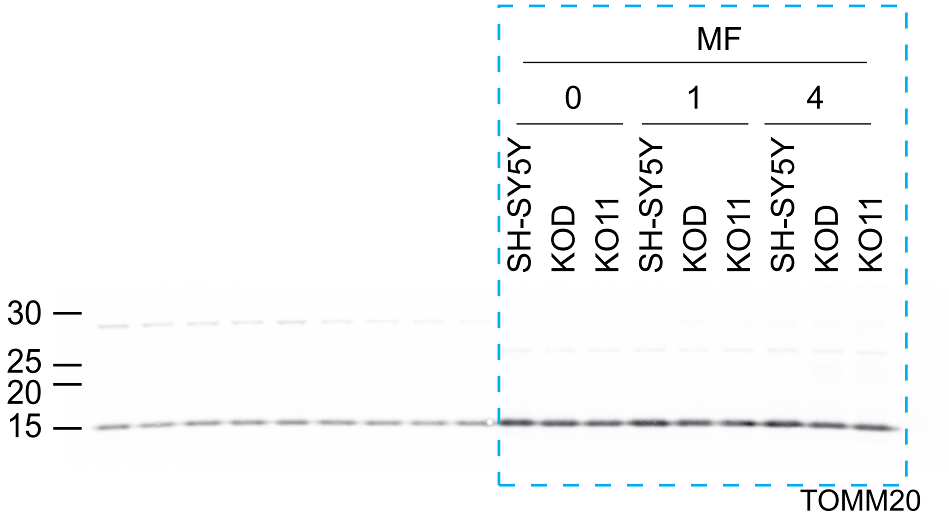

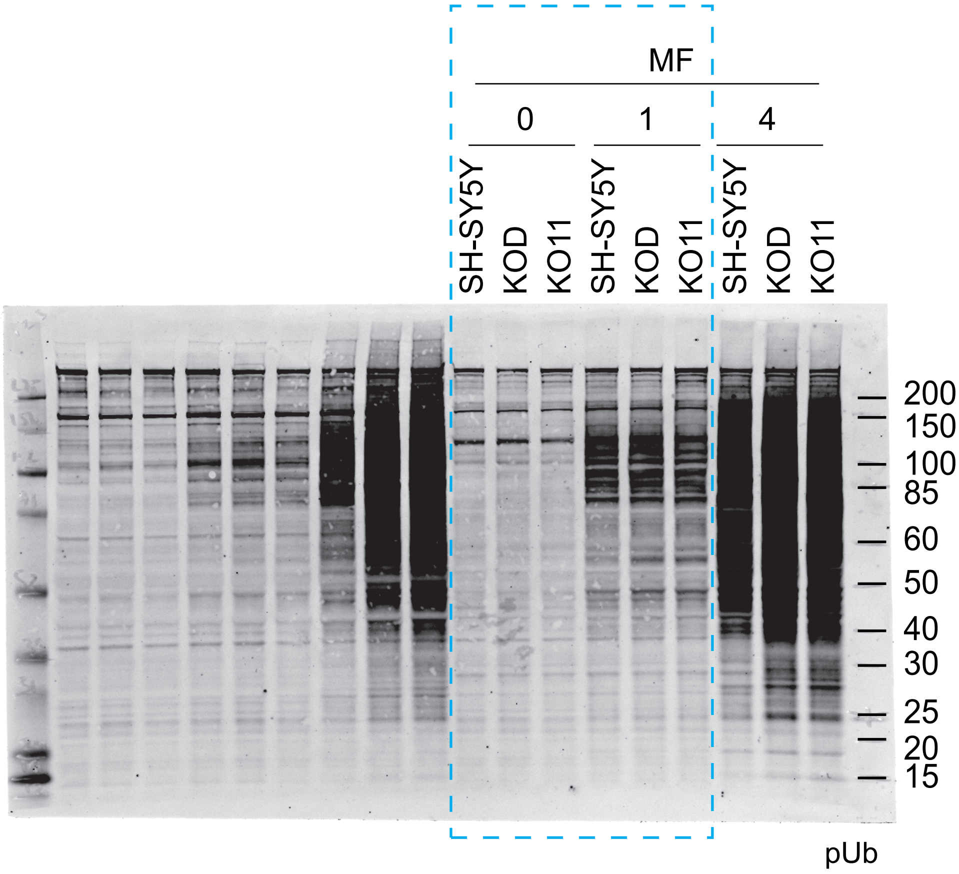

Supplement: Supplementary file 5 [file LSA-2020-00768_SdataFS2.1.pdf]
