# Supplementary material for: USP30 sets a trigger threshold for PINK1–PARKIN amplification of mitochondrial ubiquitylation
Source: Life Sci Alliance. 2020 Jul 7;3(8):e202000768. doi: 10.26508/lsa.202000768 (PMC7362391; doi:10.26508/lsa.202000768)
Supplement: Supplementary file 10 [file LSA-2020-00768_SdataF6.1.pdf]

Source Data Figure 6A

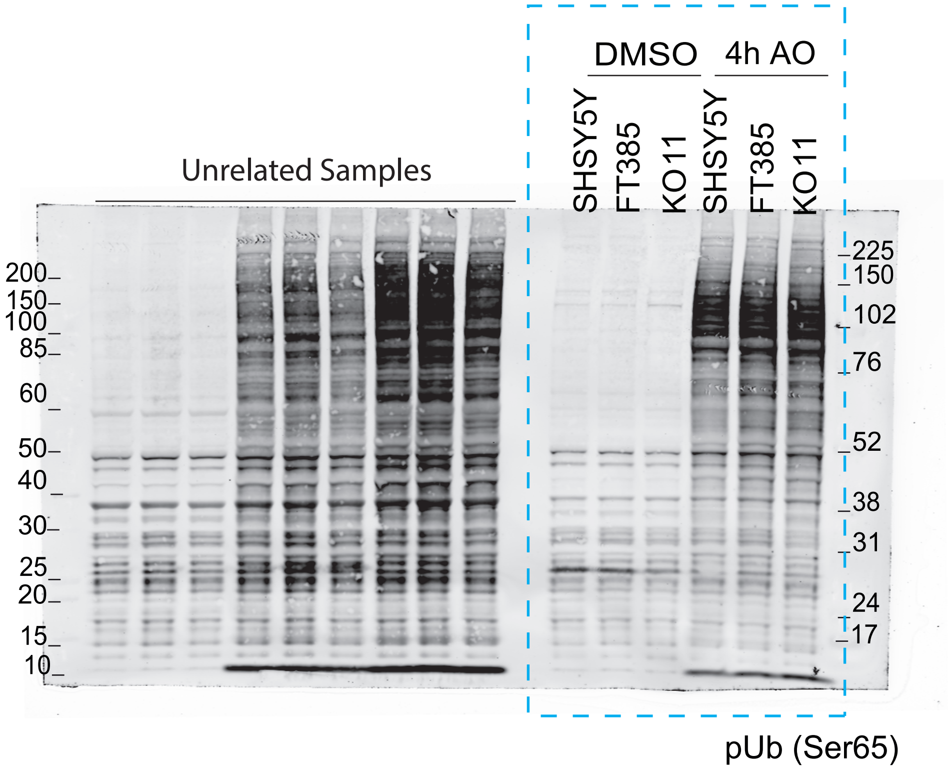

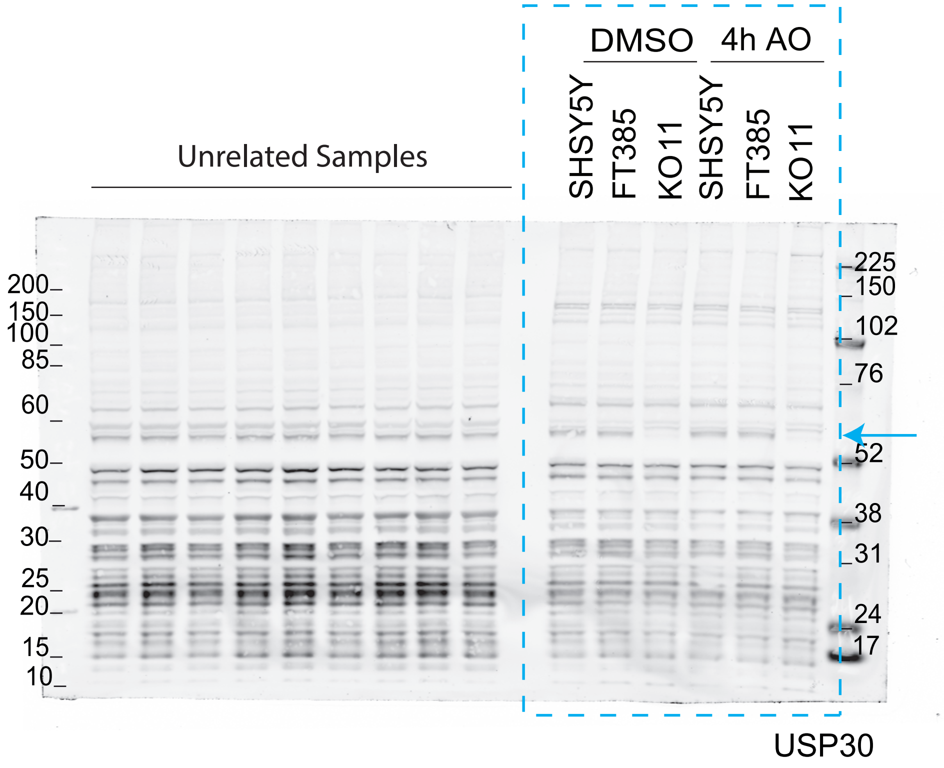

Source Data Figure 6A Cont.

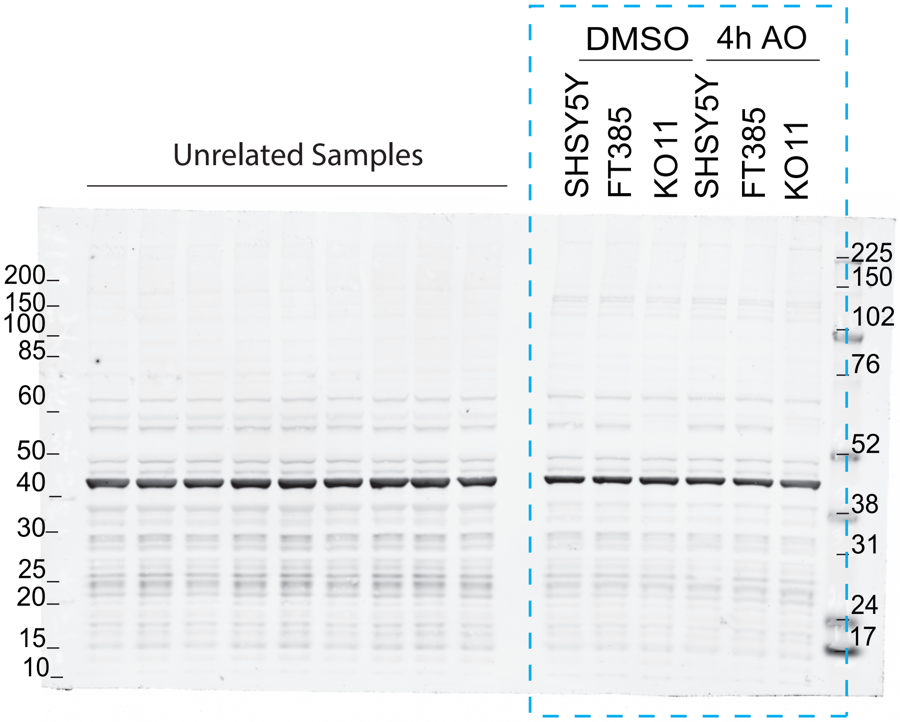

Actin (over USP30)

Source Data Figure 6A Cont.

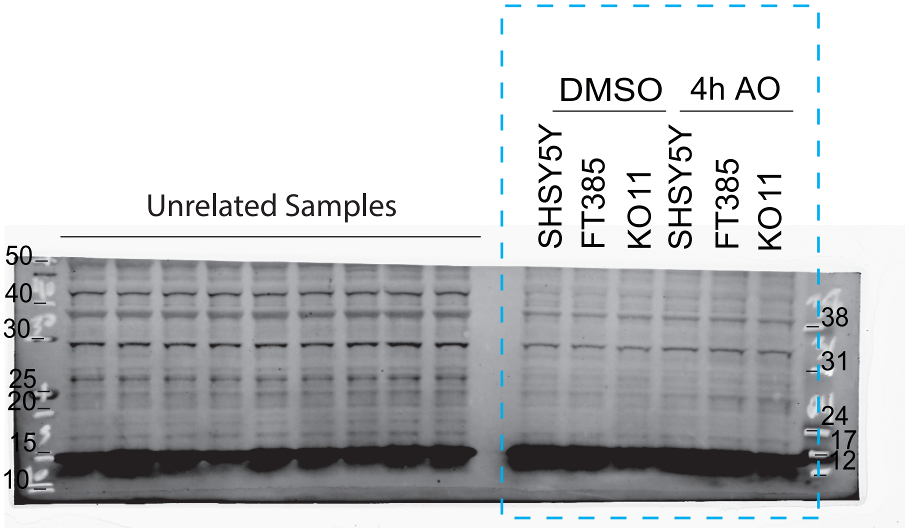

TOM20 (Higher Exposure)

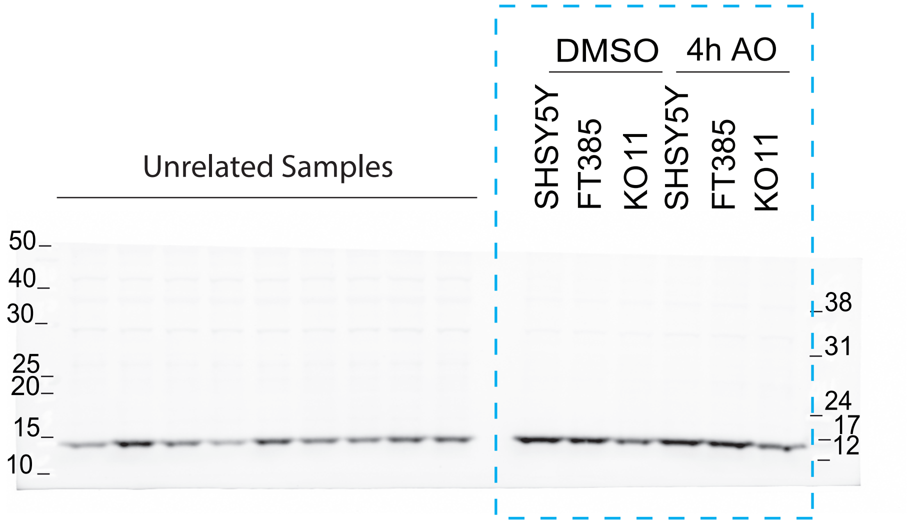

TOM20

Source Data Figure 6A Cont.

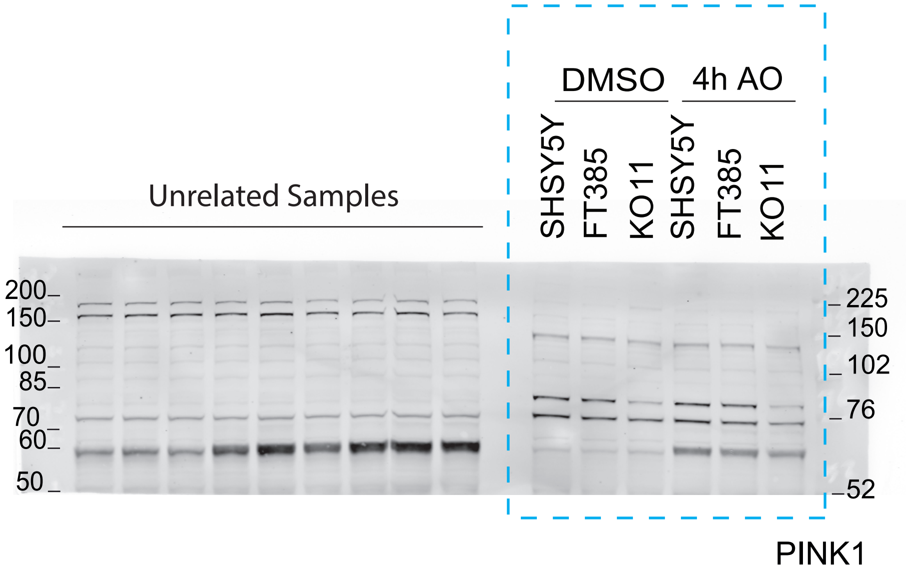

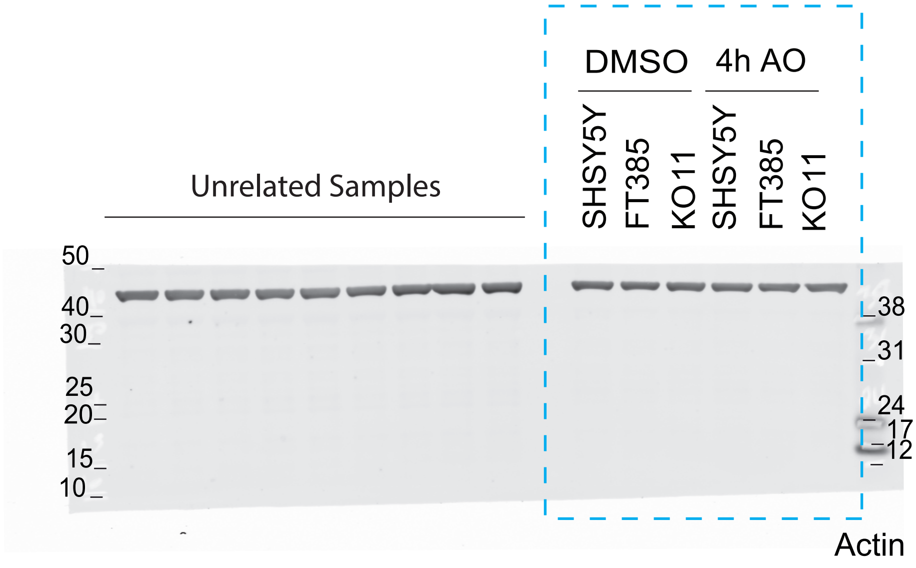

Source Data Figure 6C

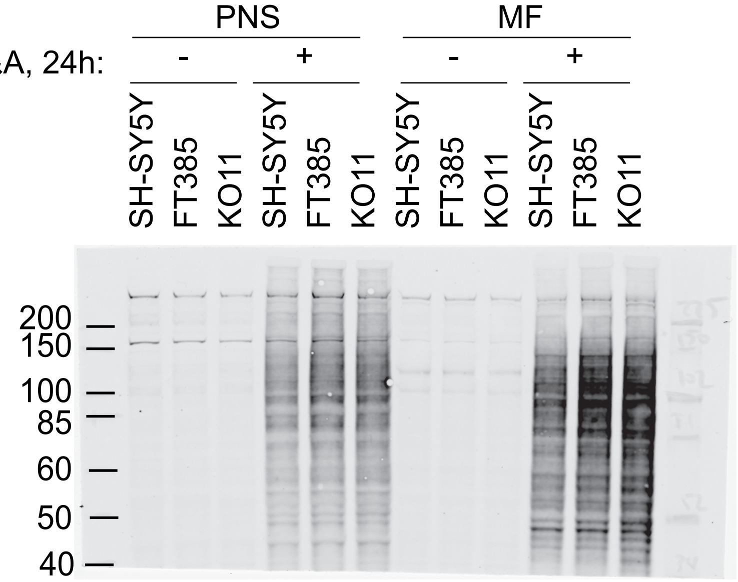

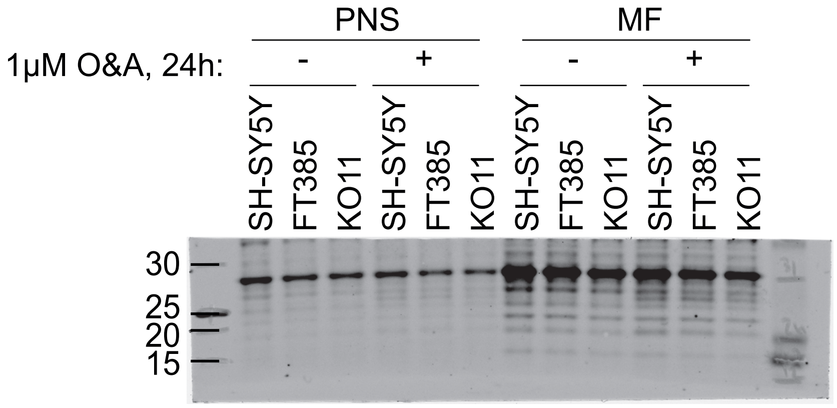

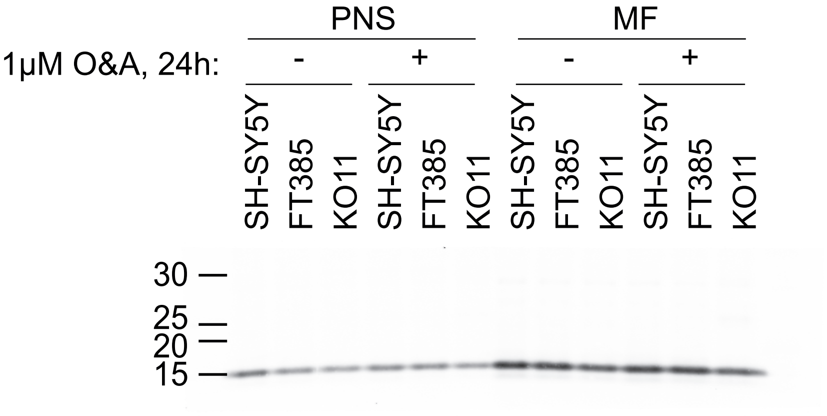

Source Data Figure 6C Cont.

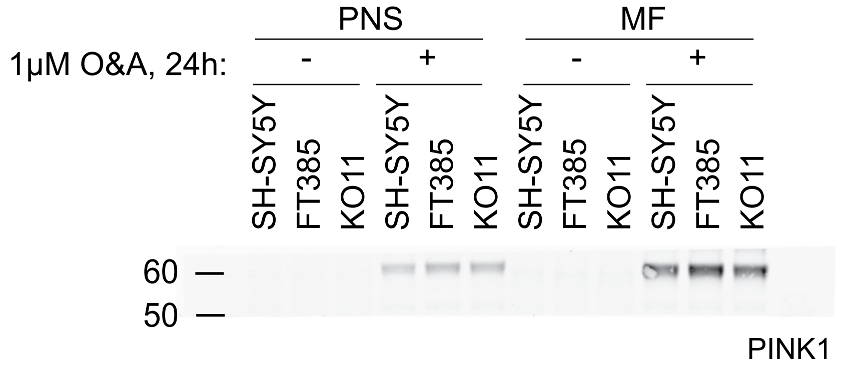

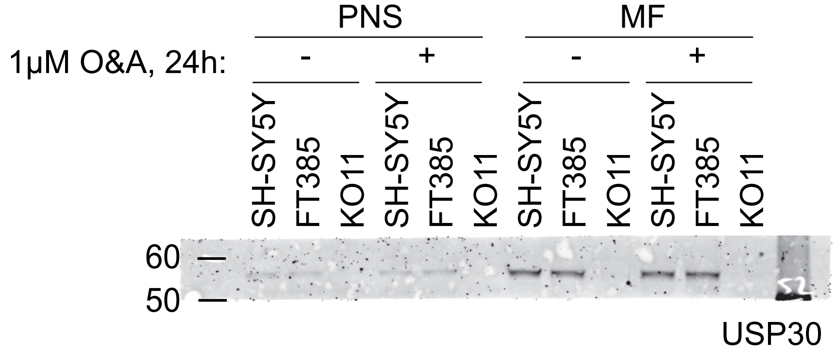

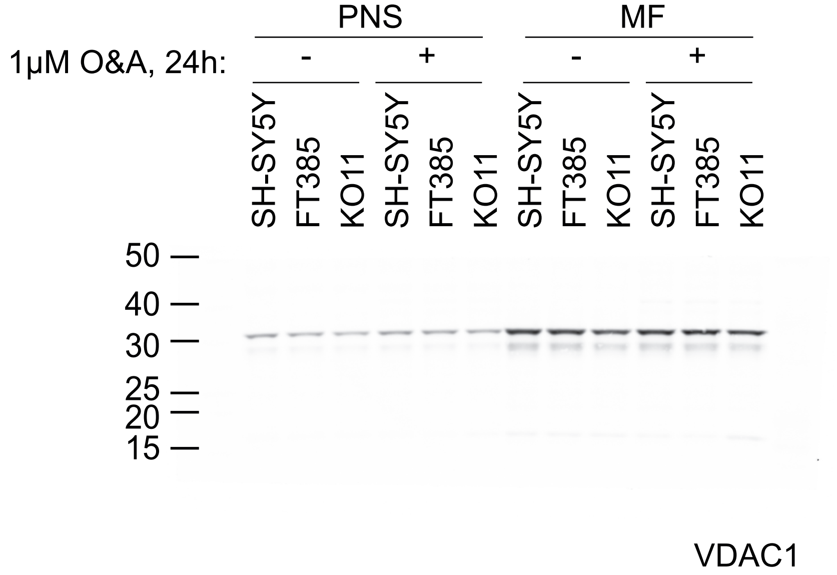

Source Data Figure 6D

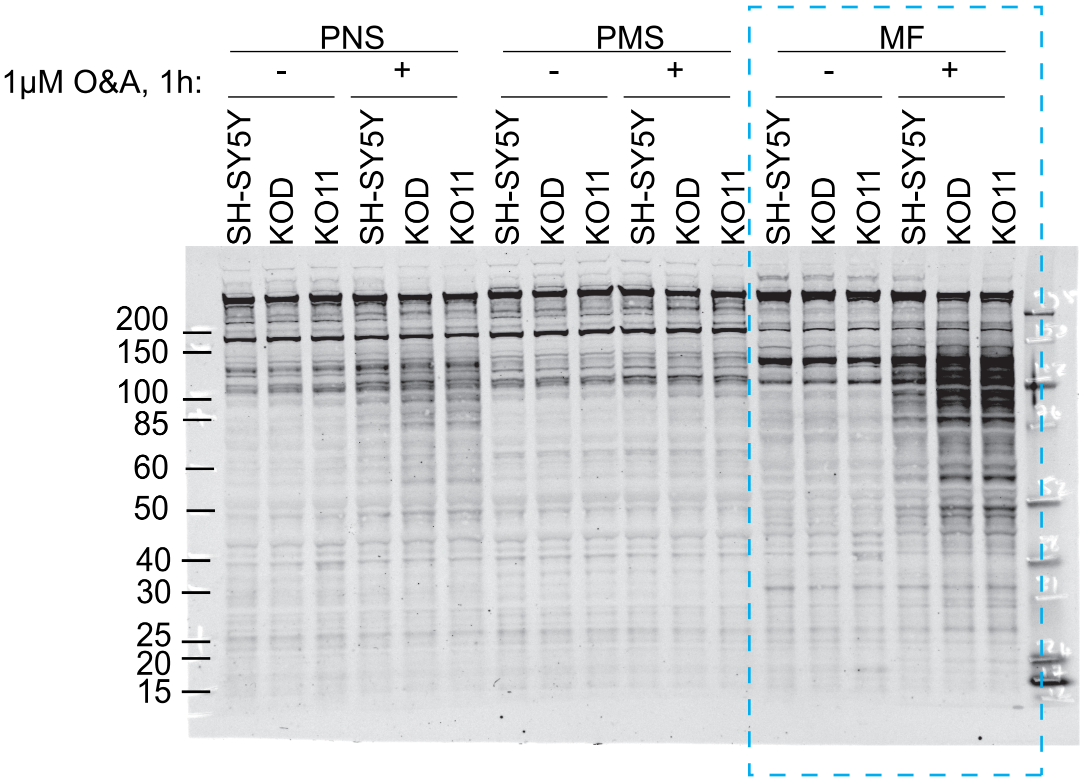

pUb (Ser65)

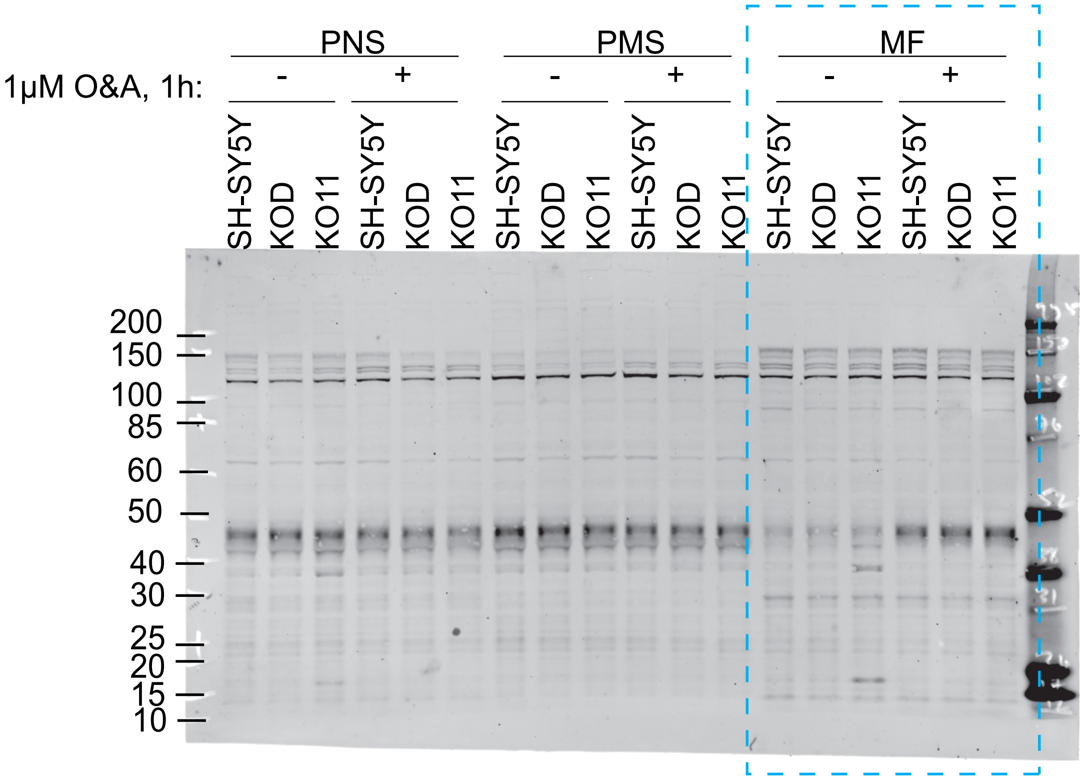

PARKIN

Source Data Figure 6D Cont.

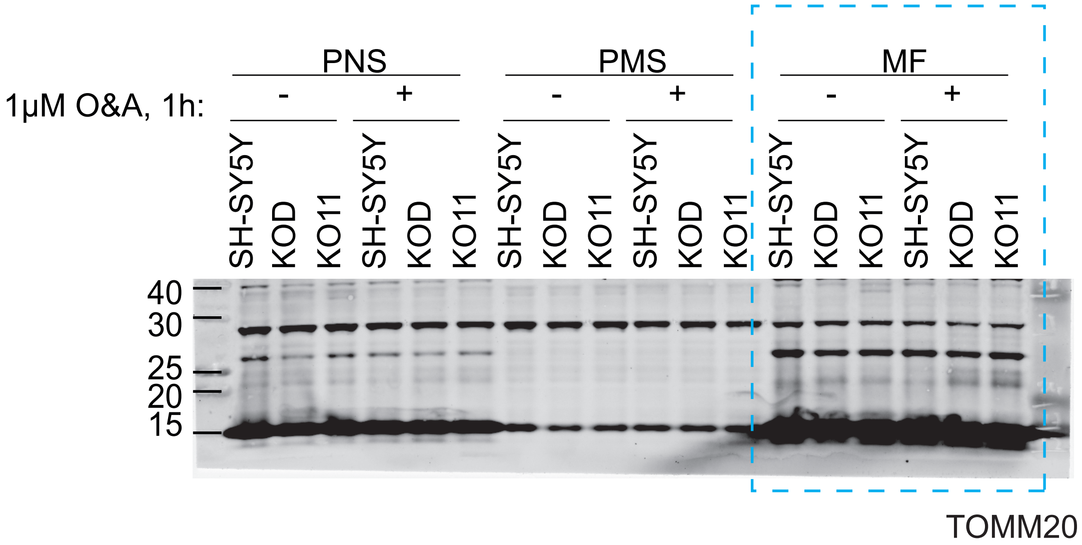

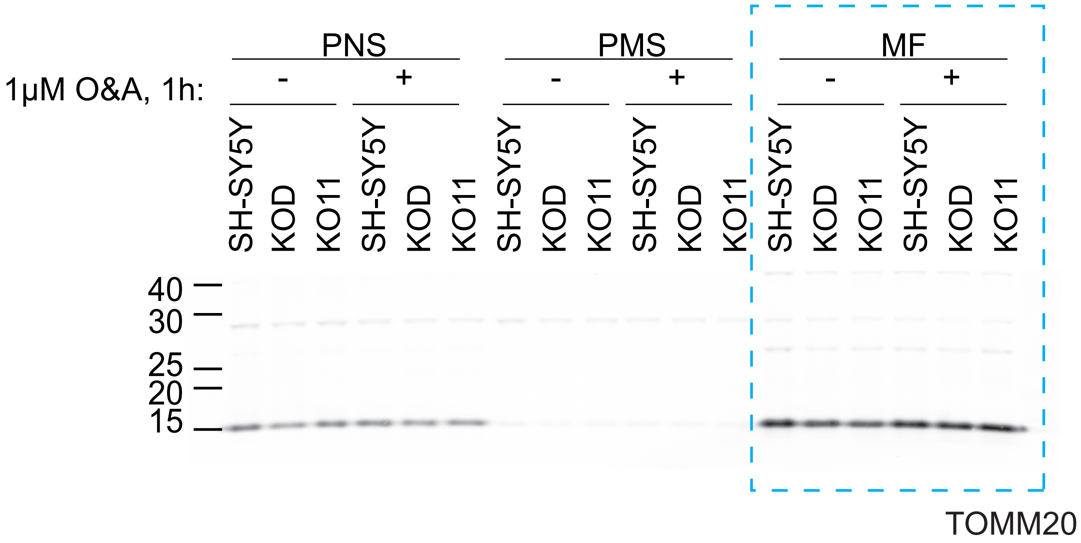

Source Data Figure 6D Cont.

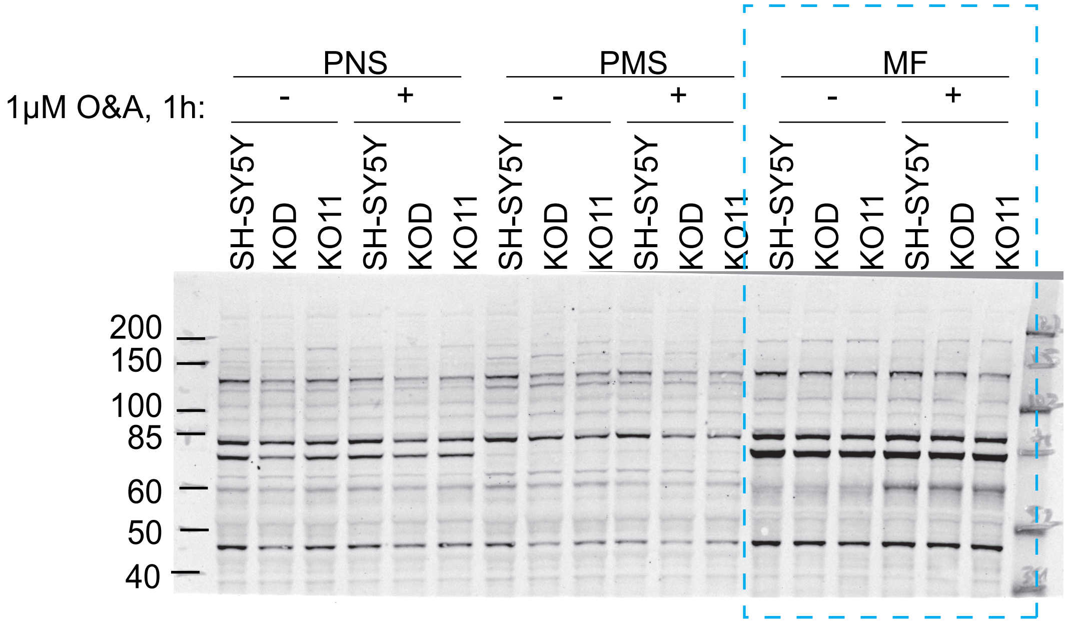

PINK1

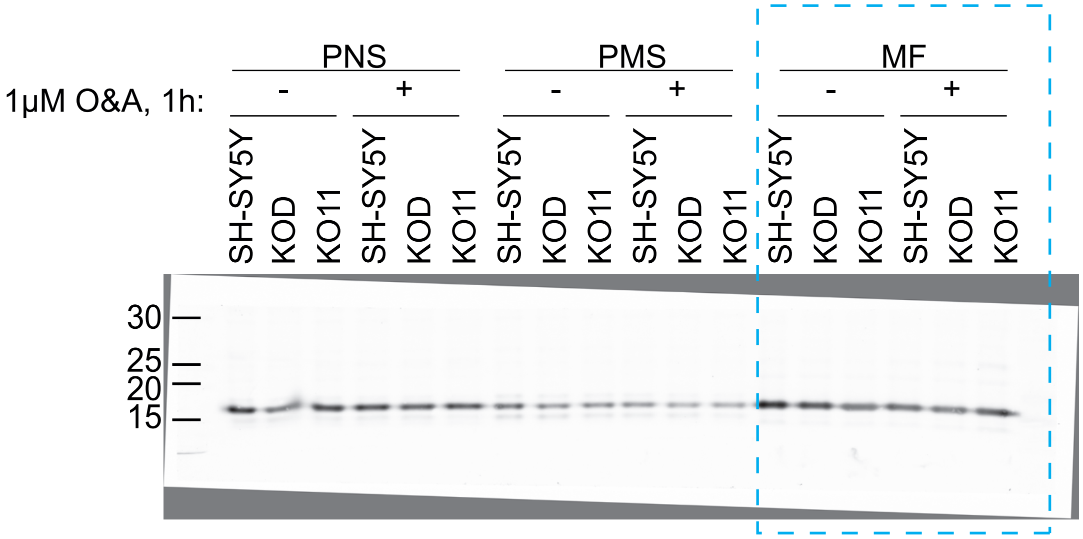

FIS1

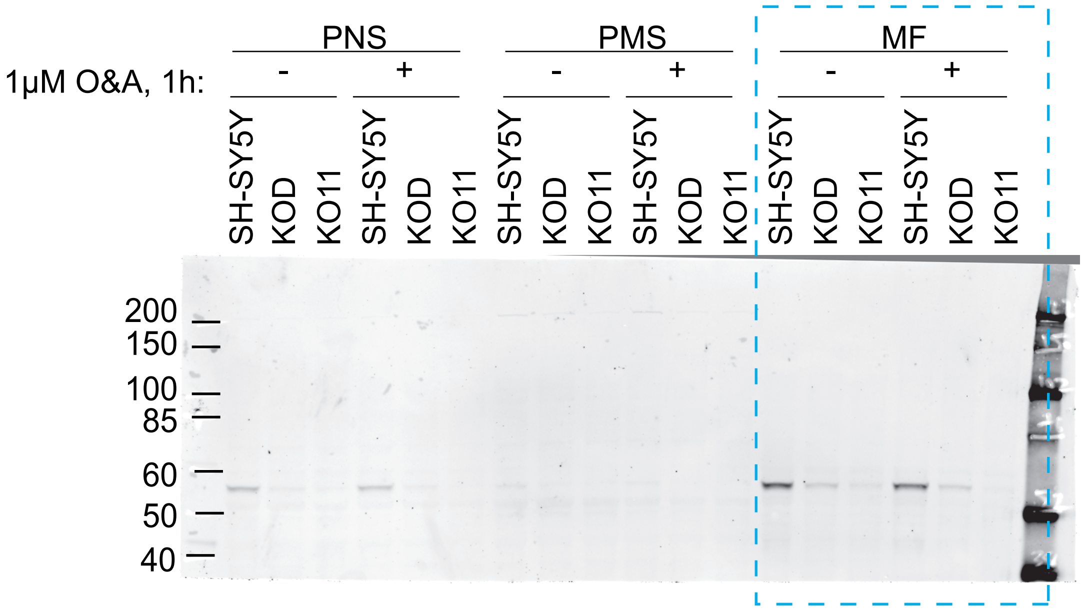

USP30
