# Supplementary material for: USP30 sets a trigger threshold for PINK1–PARKIN amplification of mitochondrial ubiquitylation
Source: Life Sci Alliance. 2020 Jul 7;3(8):e202000768. doi: 10.26508/lsa.202000768 (PMC7362391; doi:10.26508/lsa.202000768)
Supplement: Supplementary file 12 [file LSA-2020-00768_SdataFS4.pdf]

Source Data Figure S4A
Same samples as Fig3C

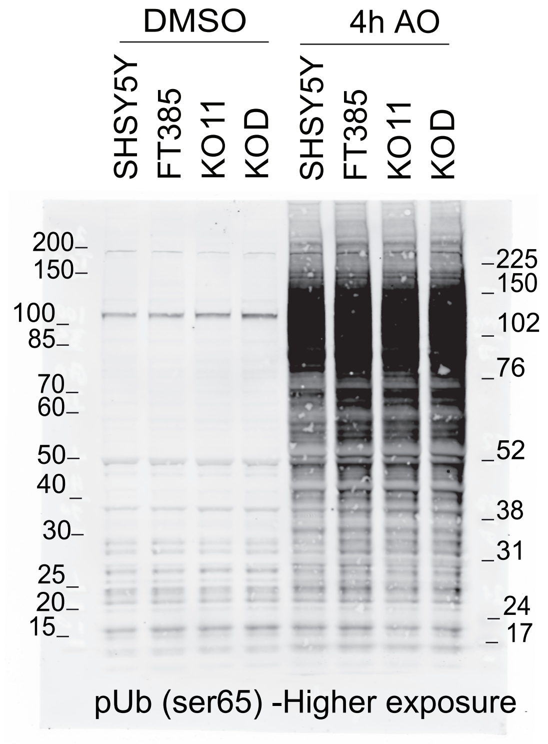

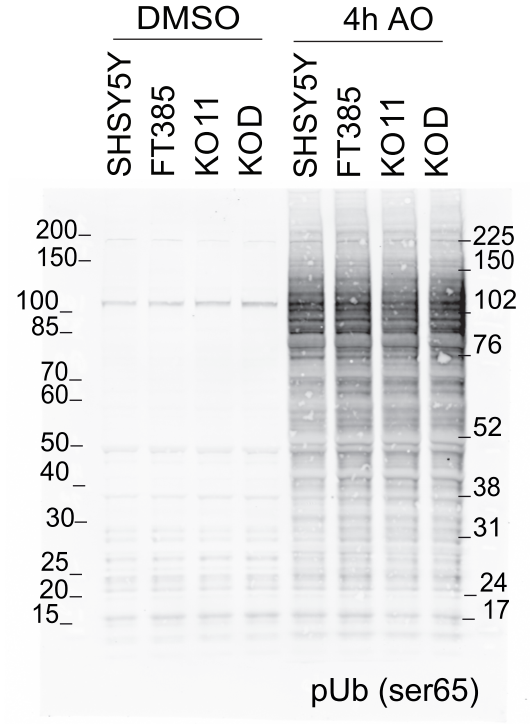

Source Data Figure S4A Cont.
Same samples as Fig3C

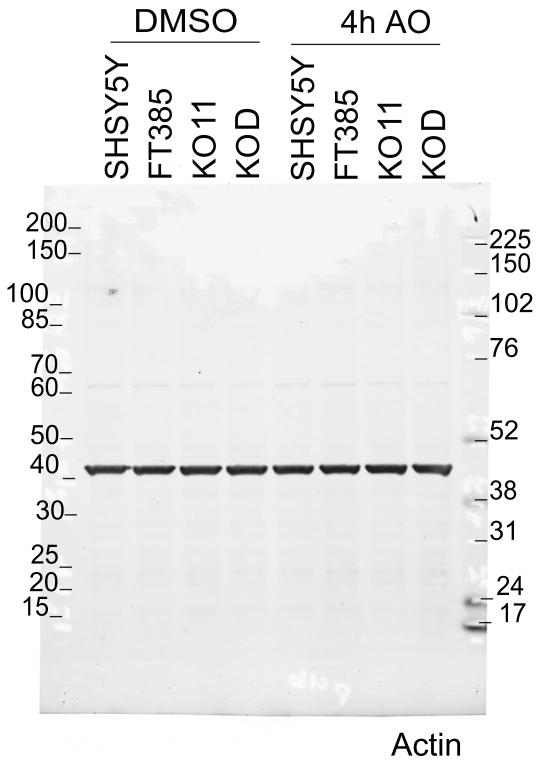

Source Data Figure S4A Cont.
Same samples as Fig3C

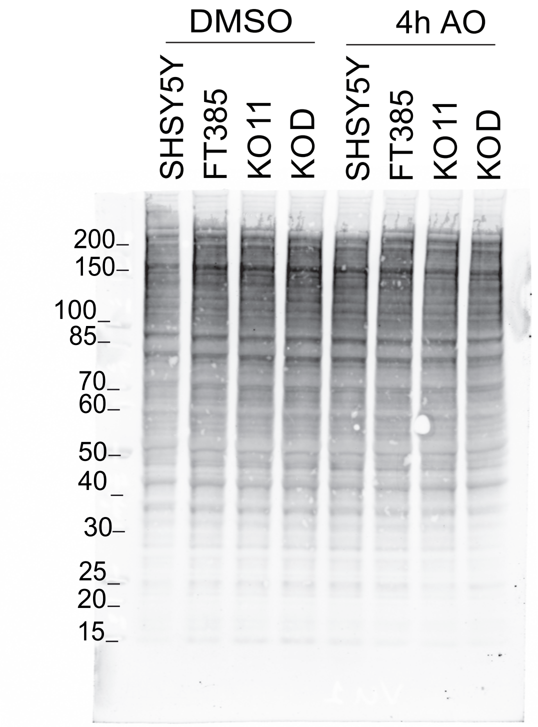

VU1

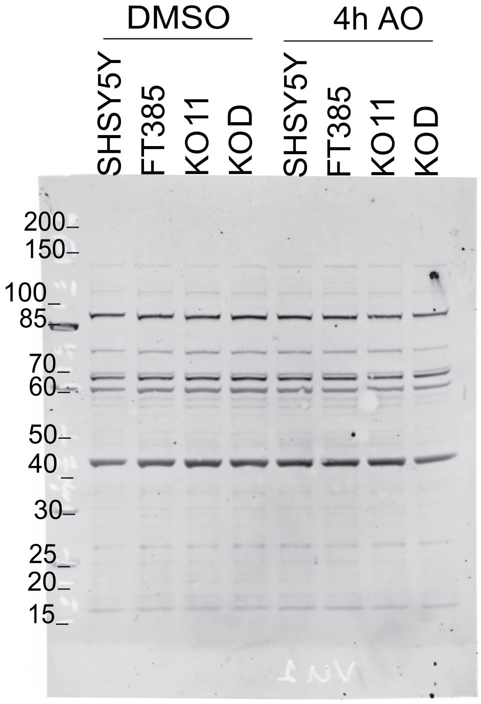

actin

Source Data Figure S4B
Same samples as Fig6A

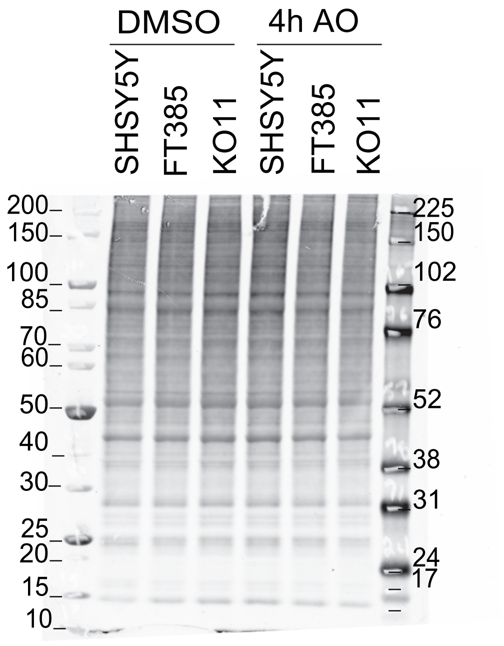

Ubiquitin (VU1)

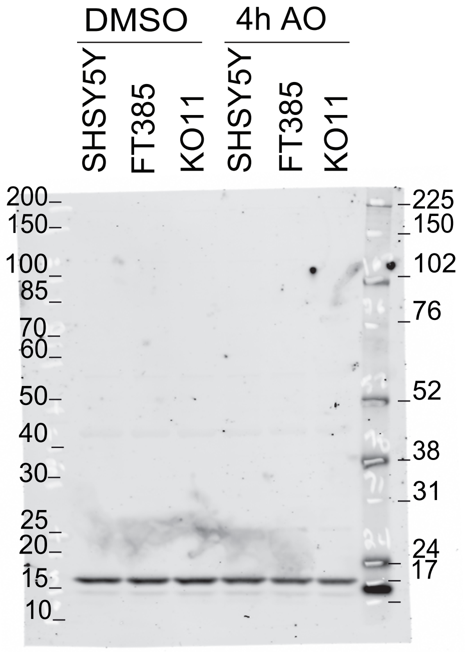

FIS1

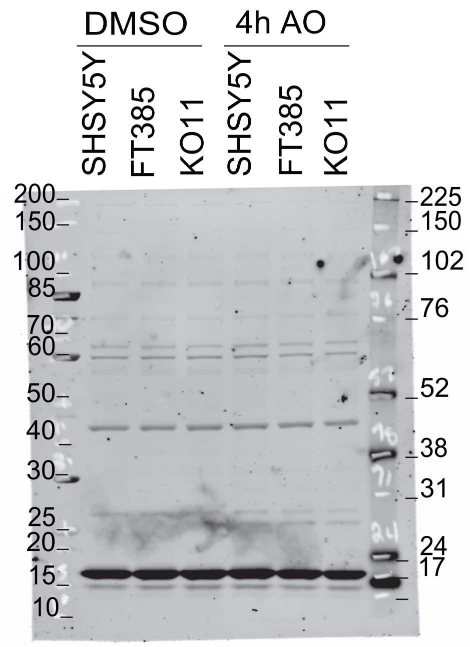

Actin (Over Fis1)
